# Validating the contributions of stem and root traits to barley lodging in western Canada

**DOI:** 10.3389/fpls.2025.1547207

**Published:** 2025-08-28

**Authors:** Michael W. Taylor, Céline A. M. Ferré, Shengjian Ye, Xuan Yang, J. Allan Feurtado, Aaron D. Beattie

**Affiliations:** ^1^ Department of Plant Sciences, College of Agriculture and Bioresources, University of Saskatchewan, Saskatoon, SK, Canada; ^2^ Aquatic and Crop Resource Development, National Research Council of Canada, Saskatoon, SK, Canada

**Keywords:** barley, lodging, root system architecture, crown root angle, solidity, plant height

## Abstract

Lodging caused by stem buckling or root anchorage failure results in significant economic losses each year globally due to increased disease load, downgrading of quality, and yield loss. To increase lodging resistance in western Canadian barley, a study was undertaken to identify traits associated to lodging score. Mechanical and architectural traits encompassing whole plant, stem and root features of 13 spring barley genotypes, representing a diversity of classes, height, and lodging resistance, were evaluated at six field locations over three years. Correlation analysis identified plant height, internode length, crown root angle and root system solidity as traits with the largest influence on lodging (r = 0.37, 0.27, -0.32 and 0.62, respectively). A structural equation model (SEM) was created to further evaluate which traits had direct or indirect influence on barley lodging. The best fitting SEM included nine traits that captured aspects of the whole plant, stem and root system. Plant height (effect estimate = 0.46) and root system solidity (0.14) showed a direct influence on lodging, while root angle had both direct (-0.21) and indirect (via height) influence on lodging. Stem strength, stem outer diameter, and stem volume had indirect effects on lodging through height, while root network area, convex area and total root length had indirect effects on lodging through root system solidity. The three traits that directly influenced lodging in both correlation analysis and SEM, plant height, root angle, and root system solidity, displayed moderate to high heritability (0.85, 0.78 and 0.57, respectively), thus making them suitable for breeding selections. Collectively, this study confirmed the role of plant height and root angle in lodging, identified root system solidity as a novel trait associated to barley lodging resistance, and revealed root imaging as a new screening tool to help breeders select for, and improve, lodging resistance in the absence of observable field lodging.

## 1 Introduction

Barley (*Hordeum vulgare* L.) is the 4^th^ most grown field crop globally, with 148 million tons of annual production (United States Department of Agriculture, 2025) and an annual export trade value of $9.5 billion USD over the past decade (World Bank, 2025). In Canada, barley is the 3^rd^ largest crop by area, with an average of 3.0 million hectares seeded over the past five years (Statistics Canada, 2025a). It is estimated that barley contributes over $14.4 billion CND annually to the Canadian GDP through farm-gate sales, exports of malt and barley, taxation on alcohol, and employment creation (LMC International, 2023).

Lodging is a detrimental phenomenon that occurs globally throughout all barley-growing regions with yield losses estimated between 3-40% depending on the growing season, growth stage at which lodging occurs and cultivar (Dyson et al., 1984; Laidig et al., 2022; Stanca et al., 1979). This can result in significant financial losses to the industry due to the combination of yield loss, increased disease load, harvest difficulty, and negative impacts on grain quality (Berry et al., 2004). For example, annual losses in the UK due to lodging across all cereal crops was estimated at ₤60-200 million (Baker et al., 2014). In Canada, there was an average price difference of $55 per ton between malt and feed barley price over the past decade (Statistics Canada, 2025b). Thus, downgrading barley from malt to feed due to increased disease load or decreased quality as a result of lodging would result in a loss of $200 per hectare.

Berry et al. (2006) described lodging as “buckling in any part of the stem (stem lodging) or failure of the root-soil anchorage system (root lodging)” which results from the interaction of environmental, agronomic, and genetic factors. The primary environmental factors typically associated with lodging are wind and precipitation, but disease, insects, and soil composition are also contributing elements (Bayer Crop Science, 2021; Gavloski, 2019; Lynch, 1995; Ma and Yamaji, 2006; Niu et al., 2016; Sterling et al., 2003). For example, Niu et al. (2016) observed that sustained wind speeds of 14.9 m/s produced 20% lodging in wheat, while wind gusts of 26.5 m/s could cause 80% lodging. When heavy rain was introduced a wind speed of only 8.4 m/s was required to cause severe lodging (Niu et al., 2016). Following rain events, the resulting increase in soil moisture can impact lodging by affecting root anchorage strength. An increase in soil moisture from 17% to 26% decreased soil shear strength and the peak load that wheat roots could resist before root anchorage failure occurred (Easson et al., 1993).

Agronomic practices such as fertilizer rates, seeding rates and the use of plant growth regulators also influence lodging. Higher nitrogen rates cause an increase in the shoot to root ratio which leads to greater lodging. At low nitrogen application rates (0.09 g nitrogen per plant) the shoot to root weight ratio was observed to be 2:3, but increased to 3:1 at higher nitrogen rates (0.46 g nitrogen per plant) (Grossman and Rice, 2012). O’Donovan et al. (2011) observed that barley lodging was more prevalent as nitrogen increased across five application rates ranging from 0-120 kg/Ha. Increasing soil potassium has been documented to reduce lodging in corn (Melis and Farina, 1984), oat (Casserly, 1956), rice (Liao et al., 2023) and wheat (Beaton and Sekhon, 1985), possibly by increasing stem wall strength which has been observed in some studies (Li et al., 2022; Melis and Farina, 1984). Seeding rates influence competition among plants and lower seeding rates have been reported to limit competition which leads to increased stem strength, decreased height and less lodging (Hobbs et al., 1998). Higher seeding rates in barley were shown to increase height (Tidemann et al., 2020) and when combined with later seeding dates caused an increase in lodging rate (O’Donovan et al., 2012).

Plant growth regulators (PGRs) are another option to manage lodging in cereal crops through reduction of plant height and increasing stem diameter and strength (Jung, 1984). PGRs, such as chlormequat chloride and trinexapac-ethyl, inhibit gibberellin synthesis which reduces cell elongation and ultimately internode length. Despite the benefits of PGRs, issues associated with variety- and environment-specific efficacy (Rajala and Peltonen-Sainio, 2002; Tidemann et al., 2020; Wiersma et al., 1986), delayed maturity (Ma and Smith, 1992a) and decreased grain weight (Ma and Smith, 1992b; Tidemann et al., 2020) are undesirable attributes associated with their use.

There are several important plant traits with a strong connection to lodging resistance, including height, stem strength and root plate spread (Berry et al., 2006) that can be targeted by plant breeders. Plant height impacts lodging in two critical ways. Firstly, a tall plant is exposed to greater wind force which increases the tendency for stem failure or uprooting (Berry et al., 2006), and secondly, the leverage created by the weight of the head, which can cause stem bending, is greater for a tall plant (Kristensen et al., 2016; Kuczyńska et al., 2013). Stem strength represents the ability of the stem (or internode) to resist forces, such as wind, and is influenced by both physical properties and composition. For example, a large basal internode diameter and thicker stem walls have been associated with higher stem strength in wheat (Yao et al., 2011). Similarly, the *STRONGCULM2* (*SCM2*) gene in rice was demonstrated to increase stem wall thickness and stem diameter which imparted better lodging resistance (Ookawa et al., 2010). With regards to chemical composition, increased expression of enzymes involved in lignin synthesis as well as increased lignin deposition have been associated with stronger lodging resistance (through increased stem strength) in wheat and barley (Begović et al., 2018; Zheng et al., 2017).

Root characteristics that influence lodging are less understood due to the difficulty of studying below-ground traits, however, traits like root plate spread, angle, and volume have been shown to increase anchorage strength and subsequently prevent lodging. For example, Wu and Ma (2018) identified that root volume was positively associated with root anchorage strength in canola. Crook and Ennos (1994) indicated that root anchorage strength (and thus lodging resistance) was dependent upon the root angle and strength of the crown roots. Similarly, root plate spread in spring and winter wheat was correlated with root anchorage across multiple environments around the world (Berry et al., 2003c; Dreccer et al., 2020; Piñera-Chavez et al., 2016a). Root growth angle and volume have also been reported in maize to be important for root-lodging resistance (Hebert et al., 1992; Zhang et al., 2022).

Within the context of the traits listed above, the development of lodging models for wheat and barley have been foundational for lodging research and identified both stem and root lodging as important determinants for overall standability (Baker et al., 2014; Berry et al., 2006, 2007). In these models, wheat (or barley) is represented by two masses, the ear and root crown, connected through a weightless stem (Baker et al., 2014). In the model for barley, Berry et al. (2006) demonstrated that stem diameter of the middle internodes had the largest effect on stem lodging risk, while changes to ear area, drag coefficient, plant height, shoot natural frequency, and stem strength had moderate influence. For wheat, stem radius was identified as most important, followed by stem failure stress and ear area. However, in wheat, failure of the stem has been modeled and demonstrated experimentally to occur close to the soil surface (Berry et al., 2003a, c). For root lodging, anchorage failure in barley has been shown to be like wheat, where the most important traits were spread of the root plate and tiller number with structural rooting depth and ear area contributing less (Berry et al., 2003c, 2006).

More recently, structural equation modeling has been applied to evaluate and quantify the causal relationships among traits influencing lodging-related traits such as stem strength. In the structural equation model (SEM) developed by Li et al. (2023), flag leaf net photosynthetic rate, stem chemical components (lignin, cellulose, soluble sugars), filling degree, and stem wall thickness were identified as key components of stem breaking strength in wheat. Structural equation modeling is a multivariate analysis method that allows for the examination of hypothetical models that describe large, intercorrelated and complex systems (Lamb et al., 2011) by analyzing the direct and indirect influences of numerous variables relevant to the system (Kline, 2015). Hypothetical models are constructed to portray causal relationships among relevant variables based on *a priori* knowledge obtained from past observations and/or relevant literature. The covariance matrix calculated among the variables in the hypothetical model is then assessed against the actual covariance matrix derived from experimental data to determine how well the model represents the data. Through an iterative process of adding or removing variables, the initial hypothetical model is altered and reassessed against the experimental data until a statistically significant, good fitting model is obtained (Lamb et al., 2011). While SEMs and correlation analysis both describe the relationship between variables, the multivariate aspect of SEM provides additional insight as to how a group of causal traits interact to influence the primary trait of interest.

The most common and simple lodging assessment method is by visual scoring of field plots (Berry et al., 2004). While this is traditionally done by eye, the use of unoccupied aerial vehicles (UAV) carrying multispectral cameras and other sensors has now been shown to accurately assess lodging (Chauhan et al., 2019; Sakamoto et al., 2010; Wilke et al., 2019). Visual or UAV lodging assessment is only possible when suitable environmental conditions (e.g. storm events) and management techniques (e.g. higher nitrogen application) promote lodging, however, it is often the case that lodging does not occur. Various methodologies have been used to assess lodging resistance in the absence of lodging by testing the mechanical or bending resistance at the whole plant or canopy level, or by assessing the strength of individual stems (Zhang et al., 2016; Erndwein et al., 2020). Berry et al. (2003b) designed an H-shaped push-bar device that measures the force required to displace a collection of stems from the vertical position. Other instruments which have been developed include the ‘Stalker’, a T-bar device which continuously records the resistance force of a group of plants when pushed from the vertical position, and a prostrate tester which measures the force required to push a collection of stems to 45 degrees from the vertical (Heuschele et al., 2019; Xiao et al., 2015).

Overall, the identification and continued refinement of screening methods which can be used in the absence of visual lodging will help breeders select for, and improve, lodging resistance. The present study focused on evaluating a variety of field and lab-based methods that measured mechanical and architectural features of barley to determine which plant, stem, or root traits were most impactful, as assessed through correlation analysis and SEM, to lodging resistance in western Canada. The ultimate goal of this study was to identify the best methods and traits which can be used for barley lodging selections in a breeding program.

## 2 Materials and methods

### 2.1 Plant materials

Thirteen barley genotypes representing North American and European germplasm that are adapted to western Canada were used in this study (**Table 1**). The genotypes represent a diversity of spring barley classes (i.e. malt, feed, and forage) that vary in plant height and lodging resistance.

**Table 1:** Description of the barley genotypes used in the study.

Genotype	Country of origin	Lodging rating	Type and class
AB Cattlelac	Canada	Very Good	6 Row Forage
AC Metcalfe	Canada	Poor-Fair	2 Row Malt
AC Ranger	Canada	Poor	6 Row Forage
Amisk	Canada	Very Good	6 Row Feed
CDC Austenson	Canada	Good	2 Row Feed
CDC Bow	Canada	Very Good	2 Row Malt
CDC Maverick	Canada	Poor	2 Row Forage
CDC Meredith	Canada	Poor-Fair	2 Row Malt
CDC PolarStar	Canada	Poor-Fair	2 Row Malt
Laureate	Europe	Good	2 Row Malt
Oreana	USA	Fair-Good	2 Row Malt
Sirish	Europe	Very Good	2 Row Malt
TR15151	Canada	Very Good	2 Row Malt

### 2.2 Field trial design

Field trials were completed in the summers of 2020-23 as randomized complete block designs with three replications. The 2020 trials were grown at Saskatoon, SK (irrigated; 52°08’N, 106°36’W; 486m above sea level; dark brown, fine texture clay-clay loam soil) and Waldheim, SK (52°37’N, 106°39’W; 549m above sea level; black medium texture loam soil), 2021 trials were grown at Saskatoon, SK, 2022 trials were grown at Saskatoon, Kernen Crop Research Farm (KCRF; 52°09’N, 106°31’W, 486m above sea level, orthic dark brown, clay-clay loam soil) and Rosthern, SK (52°39’N, 106°20’W, 505m above sea level, black, medium texture loam soil), and 2023 trials were grown in Saskatoon, KCRF, and Rosthern. All plots were seeded at a rate of 300 plants/m^2^ and a depth of 3.8 cm. Plots were 1.22 m x 3.66 m (4.47 m^2^). The seed was treated with 325 ml/100 kg seed of Raxil Pro (active ingredients: tebucanzole, prothioconazole and metalaxyl; Bayer Crop Sciences). Fertilizer was applied during seeding using a 32-23-0 blend at 56 kg/Ha (18 kg/Ha N and 14 kg/Ha P) via granular fertilizer, application rates may change based on lab soil results. At the Saskatoon, KCRF and Waldheim locations, the field was chem-fallowed the prior year, at Rosthern the prior year was sown to canola. Plots were sprayed at the Zadoks stage 13 stage with Axial Extreme IPAK (active ingredients: pinoxaden, fluroxypyr, pyrasulfotole and bromoxynil; Syngenta) at 1.24 l/hectare. Fungicides were not applied.

### 2.3 Trait data collection

A total of 18 plant, stem and root traits were measured from 2021, 2022 and 2023 Saskatoon, 2022 and 2023 KCRF, and 2022 Rosthern sites. All traits were measured at the late milk to early dough stage of development (Zadoks stages 77-83). A description of all traits is provided in **Supplementary Table 1**.

#### 2.3.1 Plant traits

Visual lodging scores were recorded at sites with differential lodging among the genotypes where a score of 1 indicated no lodging and a score of 9 indicated that the whole plot was completely lodged. Plant height (mm) was measured from soil level to the top of the head not including the awns. Force (i.e., plant bending resistance) measurements were collected with the Stalker (Heuschele et al., 2020) on two sections of the middle row of each plot. The Stalker was placed on the ground parallel to the middle row of a plot and the horizontal load-detecting bar was adjusted to half the plot’s average height. The load-detecting bar was then slowly pushed from 0° to 45° from vertical and both the force (N) and angle were recorded by the onboard computer. The number of plants contacted by the load-detecting bar was recorded and force per plant was calculated using the maximum force recorded by the Stalker.

#### 2.3.2 Stem traits

Five or six stems from the Stalker-tested sections were harvested and the second internode was cut and used for subsequent measurements. The length of the 2^nd^ internode (mm) was measured and then stem strength was measured on the internode using a custom three-point bend testing machine (designed by Dr. Scott Noble, University of Saskatchewan). The stem was placed across two fulcra which were 10 cm apart with an anvil placed above the middle of the stem. The anvil was moved at a rate of 55 mm/min until the internode failed. The peak applied force (N) was recorded and used to calculate stem strength (N·mm). Internode stem strength was calculated according to Equation 2.1.


(2.1) Ss=F·(L/4)

where F is the maximum bending force (N), that is, the force exerted at the time of breaking; and L is the length of the second internode (mm) (Gordon, 1978; Wu and Ma, 2018).

After the three-point test was completed a 1-mm cross-section was cut from the internode beside the failure point. Images of the cross-sections were taken using an inverted 18MP USB 3.0 Real-Time Live Video Microscope Digital Camera connected to an Amscope stereo microscope in 2021. In subsequent years a 3MP USB 2.0 High-speed Color CMOS C-Mount Microscope Camera was used. Images were processed using custom software (developed by R. Peters, University of Saskatchewan) to extract the minimum stem wall thickness (mm), maximum inner stem diameter (mm) and maximum outer stem diameter (mm). Stem volume (mm^3^) was calculated using the inner and outer stem diameters to obtain the radius, and internode length to obtain the length.

#### 2.3.3 Root traits

The root systems of the five or six plants sampled for the internode stem measurements were excavated through shoveling. The soil volume excavated around each plant was approximately 20 cm in diameter and 25 cm in depth. Roots were placed into water-filled tubs for 15-30 minutes to remove adhering soil followed by measurement (using a ruler and protractor) of the root systems to determine their maximum root angle (degrees) and root plate spread (mm). Root angle was measured as the greatest angle between opposing crown roots. The root plate spread (or simply root spread) was the distance between the tips of the roots used to measure crown root angles (Berry, 1998).

**Figure 1:**
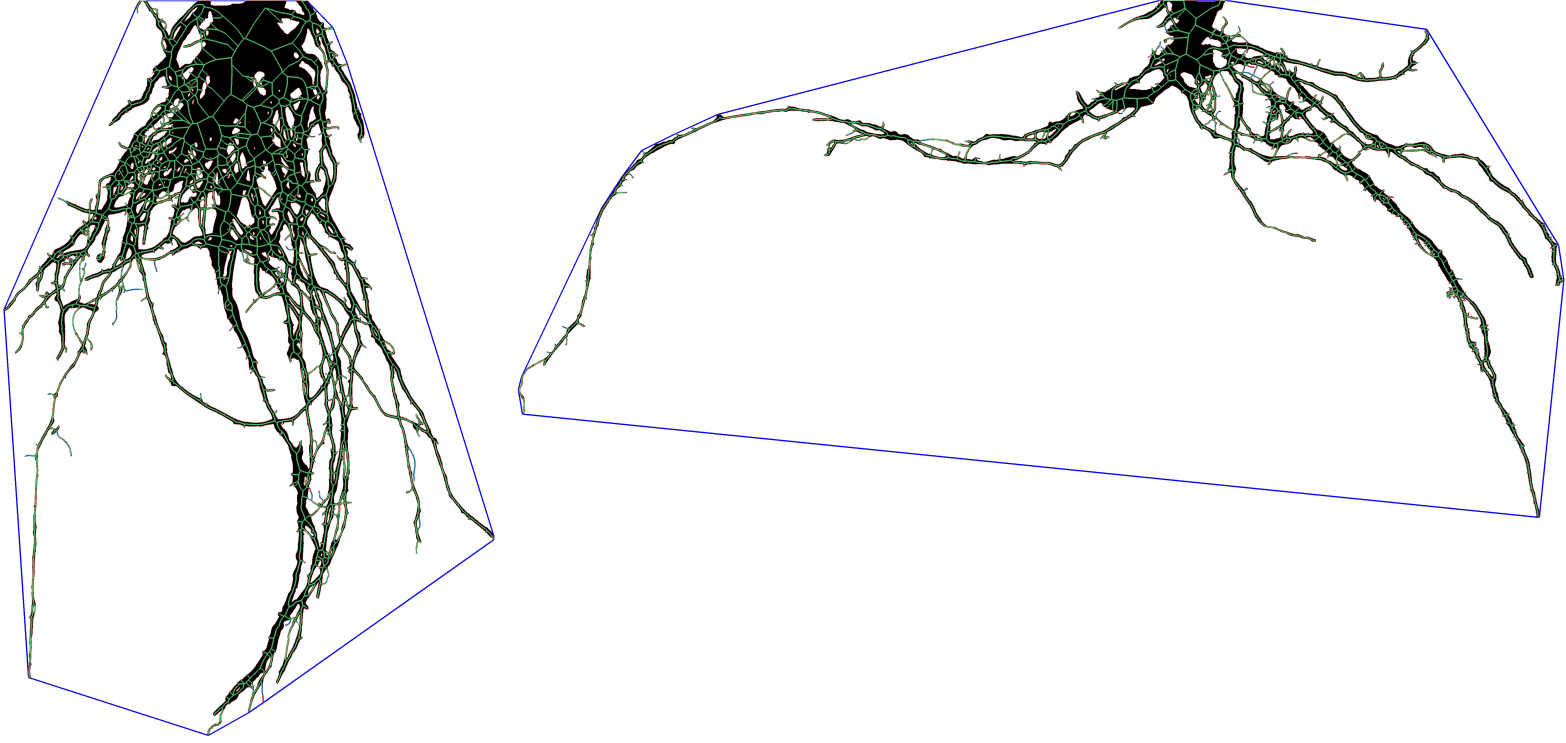
A post-processed root system image obtained from RhizoVision Explorer used to calculate root system solidity. The blue line represents the convex hull polygon that encompasses the root system, while black and green pixels represent the roots. Dividing the pixelated root area (i.e. network area) by the area encompassed by the polygon (i.e. convex area) allows the calculation of solidity. The image on the left represents a root system with high solidity while the root system on the right is one with low solidity.

Field excavated root systems were taken to a lab and imaged with a Nikon D850 DLSR camera. Images were imported into ImageJ2 (Rueden et al., 2017) where they were cropped and the contrast adjusted to improve differentiation between roots and the background medium. Images were then exported into RhizoVision Explorer (Seethepalli et al., 2021) to extract eight root traits: root diameter (mm), root number, total root length (mm), root system depth (mm), root system width (mm) network area (mm^2^), convex area (mm^2^), and solidity (mm^3^). The root trait solidity describes the ratio obtained by dividing the total root area (i.e. network area) by the area captured within a convex polygon (i.e. convex area) that encompasses the outer dimensions of the root system (**Figure 1**).

### 2.4 Statistical analysis

The effect of genotype, site-year, genotype by site-year interaction and block (i.e. replication) on each of the 18 traits were analyzed as a RCBD using a linear mixed effect model in Minitab (v. 21.2, Minitab LLC). Genotype was treated as a fixed effect while site-year, genotype by site-year interaction and blocks nested within site-year were treated as random effects. The assumptions of homogeneous variances and normal distribution of residuals were evaluated using plots of predicted values versus residuals and the Shapiro-Wilk test, respectively. A total of six site-years were used in this analysis. Significant site-year and/or genotype by site-year interactions were observed for almost all traits (**Supplementary Table 2**) so adjusted means were calculated for each genotype by site-year. Adjusted means for significant genotypic effects were separated using Tukey’s HSD *post hoc* test at a 0.05 significance level.

Four site-years produced differential lodging among the 13 genotypes from which a single adjusted mean for lodging was calculated for each genotype (using the linear mixed model described above) and used for correlation analysis to the 18 traits. A single lodging value for each genotype was used because these trials were not the same as those used to measure the 18 plant, stem and root traits and using a single value based on multiple site-years provides a more accurate indication of a genotype’s lodging resistance. As such, Pearson correlation coefficients were calculated by incorporating the single lodging value associated with each genotype into each of the six site-year datasets that contained adjusted means for each genotype for the 18 plant, stem and root traits (i.e. the lodging data was included six times in the overall dataset used for the correlation analysis). Correlation heat plots were produced with the packages ggplot2 (Wickham, 2016) and ggcorrplot (Kassambara, 2019) using the R statistical computing language (R Core Team, 2021) in the RStudio development environment (Posit Team, 2025).

Broad-sense heritability was calculated for all traits according to Equation 2.2 (Cooper et al., 1994).


(2.2) $$\text{H = }\frac{\sigma_{g}^{2}}{\sigma_{g}^{2}\;+\;\frac{\sigma_{ge}^{2}}{n_{e}}\;+\;\frac{\sigma_{e}^{2}}{n_{e}n_{r}}}$$

where H is the broad sense heritability, σ_g_^2^ is the genotypic variance, σ_e_^2^ is the error variance, $\sigma_{ge}^{2}$ is the genotype by site-year interaction, n_e_ is the number of site-years and n_r_ is the number of replications.

A structural equation model (SEM) was created with the ‘sem’ function in the ‘lavaan’ R package (Rosseel et al., 2022) to quantify the direct and indirect influence of the traits measured in this study on lodging. Structural equation models require a sample size greater than 150, thus individual plot data from the six site-years was used to meet sample-size requirements. The initial model was built based on inclusion of variables identified in this study with significant (P<0.05) correlation to lodging and additional variables shown to be relevant to lodging in other studies. Traits were added or removed in an iterative process to produce the best-fitting model. According to Xia and Yang (2018), the indices of a good fitting SEM are as follows: a chi-square P-value of >0.05, a comparative fit index (CFI) of >0.95 and a standardized root mean square residual (SRMR) of <0.08. According to Cohen (1988), estimate effects (Est) with a value of <0.1 indicate small effects, 0.3 are moderate effects and >0.5 are large effects on the variable by the predictor variable. Effect estimates were calculated by regression according to Equation 2.3.


(2.3) Y=bX+e

where Y is the predicted value, X is the predictor variable, e is the error term and b is the path coefficient. The path coefficient is calculated according to Equation 2.4.


(2.4) b=cov(X,Y)/var(X)

where cov(X,Y) is the covariance between the variable being predicted and the predictor variable and var(X) is the variance of the predictor variable (Rosseel et al., 2022). Indirect path coefficients are calculated by multiplying the path coefficients for all steps between traits (Baron and Kenny, 1986).

## 3 Results

### 3.1 Variation and heritability of plant, stem and root traits

Analysis of variance demonstrated that significant genotypic effects were observed for all plant, stem and root traits (P = 0.04 to <0.01) (**Supplementary Table 2**). Summary trait data for each genotype is provided in **Table 2** and **Supplementary Table 3**. The greatest range in trait values, expressed as a proportion calculated between the largest and smallest mean among the 13 genotypes, was observed for stem strength. A ratio of 2.90 was observed for this trait with Laureate showing the lowest value at 28 N·mm and AB Cattlelac having the highest value at 82 N·mm (**Table 2**). Other traits with a large range in values among the 13 genotypes included root spread (ratio = 2.00; Sirish = 46 mm, Amisk = 92 mm), stem volume (ratio = 1.94; Laureate = 2104 mm^3^, AB Cattlelac = 4092 mm^3^), force per plant (ratio = 1.89; Laureate = 0.08, CDC Meredith = 0.16) and convex area (ratio = 1.87; Sirish = 4575 mm^2^, Amisk = 8571 mm^2^) (**Table 2**). Root diameter showed the least range in values with a ratio of 1.15 (AC Metcalfe = 0.62 mm, Ac Ranger = 0.71 mm) (**Table 2**). Traits with similarly low trait value ranges included internode length (ratio = 1.23; Laureate and Oreana = 100, Amisk = 123), stem inner diameter (ratio = 1.27; TR15151 = 4.12 mm, AB Cattlelac = 5.23 mm), stem outer diameter (ratio = 1.28; Laureate = 6.73 mm, AB Cattlelac = 8.65 mm) and root system depth (ratio = 1.30; CDC Maverick = 90 mm, Amisk = 118 mm) (**Table 2**).

**Table 2:** Summary of adjusted means for the 18 plant, stem and root traits measured on the 13 barley genotypes used in the study.

Trait^1^	AB Cattlelac	AC Metcalfe	AC Ranger	Amisk	CDC Austenson	CDC Bow	CDC Maverick	CDC Meredith	CDC PolarStar	Laureate	Oreana	Sirish	TR15151	H^2^
Height (mm)	786 b	755 b	750 bc	723 bcd	704 bcd	750 bc	905 a	781 b	755 b	646 cd	701 bcd	634 d	736 bcd	0.85
Force Per Plant (N)	0.15 ab	0.12 ab	0.13 ab	0.13 ab	0.11 ab	0.13 ab	0.11 ab	0.16 a	0.15 ab	0.08 b	0.12 ab	0.12 ab	0.12 ab	0.81
Internode Length (mm)	112 abc	114 ab	112 abc	123 a	116 ab	104 bc	120 a	105 bc	114 abc	100 c	100 c	102 bc	104 bc	0.68
Stem Strength (N·mm)	82 a	44 def	53 bcd	69 ab	46 de	63 bc	81 a	47 cde	47 cde	28 f	31 ef	31 ef	44 def	0.82
Stem Wall Thickness (mm)	2.34 a	1.65 cd	2.13 ab	2.10 ab	1.83 bcd	1.82 bcd	1.86 bc	1.73 cd	1.56 cd	1.53 d	1.75 cd	1.67 cd	1.79 bcd	0.85
Stem Inner Diameter (mm)	5.23 a	4.38 de	4.76 bc	4.86 b	4.44 cde	4.36 de	4.70 bcd	4.37 de	4.41 de	4.34 e	4.84 b	4.48 cde	4.12 e	0.85
Stem Outer Diameter (mm)	8.65 a	7.02 de	7.90 b	7.98 b	7.26 cd	7.08 de	7.81 b	6.89 de	6.95 de	6.73 e	7.57 bc	7.16 cde	6.82 de	0.85
Stem Volume (mm^3^)	4092 a	2710 cd	3529 ab	4005 a	2970 bc	2550 cd	3686 ab	2401 cd	2631 cd	2104 d	2695 cd	2473 cd	2398 cd	0.85
Root Angle (degrees)	111 abc	87 d	113 ab	128 a	112 ab	107 bc	93 cd	87 d	87 d	83 d	106 bc	87 d	99 bcd	0.78
Root Spread (mm)	66 b-f	57 c-f	84 ab	92 a	72 a-e	77 a-c	58 c-f	52 ef	55 d-f	52 ef	71 a-e	46 f	75 a-d	0.83
Root Diameter (mm)	0.70 ab	0.62 b	0.71 a	0.67 ab	0.63 ab	0.66 ab	0.70 ab	0.65 ab	0.67 ab	0.64 ab	0.68 ab	0.69 ab	0.70 ab	0.85
Root Number	4.9 ab	5.2 ab	6.1 a	5.5 ab	4.8 ab	5.9 ab	5.1 ab	5.3 ab	5.5 ab	4.6 ab	5.6 ab	4.3 b	5.5 ab	0.77
Total Root Length (mm)	1518 a-d	1363 b-d	1845 a	1817 ab	1412 a-d	1781 ab	1363 b-d	1535 a-d	1418 a-d	1202 d	1602 a-d	1234 cd	1717 a-c	0.68
Root System Depth (mm)	104 ab	104 ab	106 ab	118 a	101 ab	102 ab	90 b	109 ab	94 ab	99 ab	107 ab	99 ab	112 ab	0.83
Root System Width (mm)	95 b-e	86 c-e	110 ab	123 a	104 a-d	109 a-c	86 c-e	83 de	88 b-e	84 de	97 b-e	76 e	91 b-e	0.83
Network Area (mm^2^)	878 ab	741 b	1141 a	1055 ab	756 b	995 ab	838 ab	849 ab	812 ab	720 b	992 ab	700 b	1006 ab	0.83
Convex Area (mm^2^)	5866 bc	5334 c	7420 ab	8571 a	6391 bc	7346 ab	4649 c	5252 c	5180 c	5027 c	6450 bc	4575 c	6493 bc	0.83
Solidity	0.161 a-c	0.149 a-c	0.172 ab	0.129 c	0.134 bc	0.158 a-c	0.187 a	0.181 a	0.178 a	0.160 a-c	0.167 a-c	0.168 a-c	0.161 a-c	0.57

Means are calculated from six site-years of data. Trait descriptions are provided in **Supplementary Table 1**.

^1^Trait means were separated using Tukey’s HSD *post hoc* test at a 0.05 significance level. Significant differences are denoted by different letters.

^2^Broad sense heritability values for each trait were calculated as per Cooper et al. (1994).

Significant differential lodging (P< 0.01; **Supplementary Table 2**) was observed among the 13 genotypes at the 2020 Waldheim and 2020, 2022 and 2023 Saskatoon sites, with AB Cattlelac, Amisk, CDC Austenson, CDC Bow, Laureate, Oreana, Sirish and TR15151 generally displaying lodging resistance while AC Metcalfe, AC Ranger, CDC Maverick, CDC Meredith and CDC PolarStar were often susceptible to lodging (**Table 3**).

**Table 3:** Summary of lodging scores obtained for the 13 genotypes from four site-years that displayed significant differential lodging.

Genotype	2020 Waldheim	2020 Saskatoon	2022 Saskatoon	2023 Saskatoon	Mean^1^
AB Cattlelac	2.4	4.7	2.2	1.9	2.8 cd
AC Metcalfe	3.6	5.0	7.9	7.9	6.1 ab
AC Ranger	8.6	7.0	7.0	6.0	7.2 ab
Amisk	1.1	1.1	2.2	2.2	1.7 d
CDC Austenson	1.9	1.1	1.6	1.7	1.6 d
CDC Bow	2.9	1.2	2.4	3.7	2.6 cd
CDC Maverick	8.9	8.1	8.6	8.7	8.6 a
CDC Meredith	8.1	7.4	8.9	8.9	8.3 a
CDC PolarStar	5.4	7.0	7.8	7.8	7.0 ab
Laureate	4.8	3.2	1.9	1.4	2.8 cd
Oreana	4.7	6.7	1.5	1.5	3.6 bcd
Sirish	1.0	1.2	1.3	1.3	1.2 d
TR15151	1.6	1.1	1.3	1.3	1.3 d

Lodging scores are based on a 1-9 scale with 1 indicating no lodging and 9 indicating the whole plot was completely lodged.

^1^Means were separated using Tukey’s HSD *post hoc* test at a 0.05 significance level. Significant differences are denoted by different letters.

Significant site-year and/or genotype by site-year interaction effects were observed for most traits (P = 0.05 to <0.01) except for internode length, root number, total root length, root system depth and solidity (**Supplementary Table 3**). Summary trait data for each site-year is provided in **Supplementary Table S4**.

Broad sense heritability estimates were moderate to high for all 18 plant, stem and root traits (**Table 2**). Among the plant traits, force per plant showed a heritability of 0.81 while plant height was determined to have a heritability of 0.85. Stem traits had heritability values that ranged from 0.68 for internode length to 0.85 for the stem size traits (stem wall thickness, inner diameter, outer diameter and volume). Finally, root traits displayed heritability values from 0.57 for solidity to 0.85 for root diameter.

### 3.2 Plant, stem and root trait correlations to lodging

**Figure 2:**
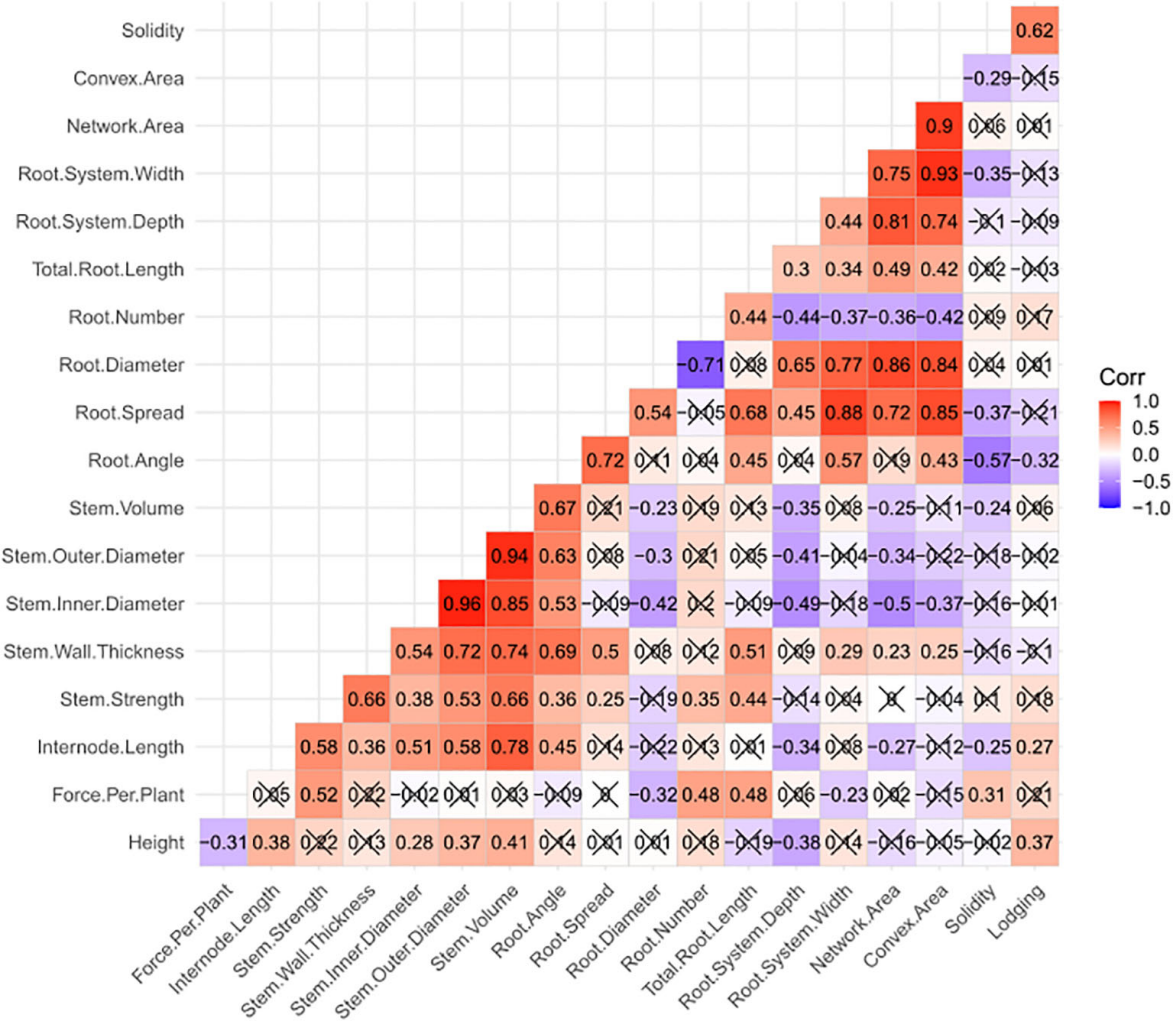
Heatmap displaying Pearson correlation coefficients among 18 plant, stem and root traits measured in the study. Lodging data is based on four site-years, while all other trait data is based on six site-years. Correlations that are not significant (P > 0.05) are crossed out.

Among the plant and stem traits, both height (r = 0.37) and internode length (r = 0.27) demonstrated significant (P< 0.05) positive correlations to lodging (**Figure 2**). Given that internode length is one component of height, it was interesting that the correlation between height and internode length (r = 0.38) was not stronger. The stem size traits (stem wall thickness, inner diameter, outer diameter and volume) were highly and positively correlated to one another (r = 0.72-0.96), with the correlation between stem inner diameter and stem thickness being moderate (r = 0.54). All stem size traits showed positive correlations to stem strength (r = 0.38-0.66), with stem wall thickness and stem volume both showing the highest influence on stem strength (r = 0.66) and stem inner diameter showing the lowest influence (r = 0.38). Similar correlation strengths were observed between the stem size traits and internode length (r = 0.36-0.78), with stem volume displaying the highest correlation and stem wall thickness the lowest. Following this pattern of correlations, stem strength showed a positive correlation to internode length (r = 0.58). Stem size traits all displayed weaker correlation to height than to internode length (r = 0.28-0.41) with stem wall thickness having a non-significant correlation. Similarly, stem strength was not significantly correlated to height.

Two root traits were also observed to have significant correlations to lodging, with root angle having a negative correlation (r = -0.32) and solidity having a positive correlation (r = 0.62) (**Figure 2**). There was also a negative correlation between root angle and solidity (r = -0.57). This was the strongest correlation with solidity, with root spread, root system width and convex area showing similar, but lower negative correlations (r = -0.29 to -0.37). It was interesting to note that network area (a component of solidity along with convex area) and root system depth were not significantly correlated to solidity. Convex area and network area were very strongly and positively correlated (r = 0.90) and shared similar strength of correlation with root number (r = -0.42 and -0.36, respectively), root diameter (r = 0.84 and 0.86, respectively), root system width (r = 0.93 and 0.75), root system depth (r = 0.74 and 0.81, respectively), total root length (r = 0.42 and 0.49, respectively) and root spread (r = 0.85 and 0.72, respectively). Root angle and root spread were strongly and positively correlated to one another (r = 0.72), and both were positively correlated to root system width (r = 0.57 and 0.88, respectively) and total root length (r = 0.45 and 0.68, respectively). In addition, root spread was positively correlated with both root system depth (r = 0.45) and root diameter (r = 0.54). Root system width and depth showed a moderate positive correlation to one another (r = 0.44), and both similarly influenced root characteristics including total root length (r = 0.34 and 0.30, respectively), root diameter (r = 0.77 and 0.65, respectively), and root number (r = -0.37 and -0.44, respectively). Finally, root number was moderately and positively correlated to total root length (r = 0.44) and negatively correlated to root diameter (r = -0.71).

Correlations were also observed between root traits and above-ground traits, however, these will not be mentioned as the basis of such correlations are less clear, perhaps reflecting an underlying genetic basis, such as pleiotropy. However, significant correlations involving the four traits directly correlated to lodging (i.e. height, internode length, root angle and solidity) are worth noting. Root angle was observed to have moderate to strong positive correlations with internode length (r = 0.45), stem strength (r = 0.36) and the four stem size traits (r = 0.53-0.69). Solidity was also significantly correlated to internode length (r = -0.25) and stem volume (r = -0.24), but also with force per plant (r = 0.31). Height and internode length both showed a negative correlation with root system depth (r = -0.38 and -0.34, respectively), while internode length was also negatively correlated to root network area (r = -0.27).

### 3.3 A structural equation model for lodging

**Figure 3:**
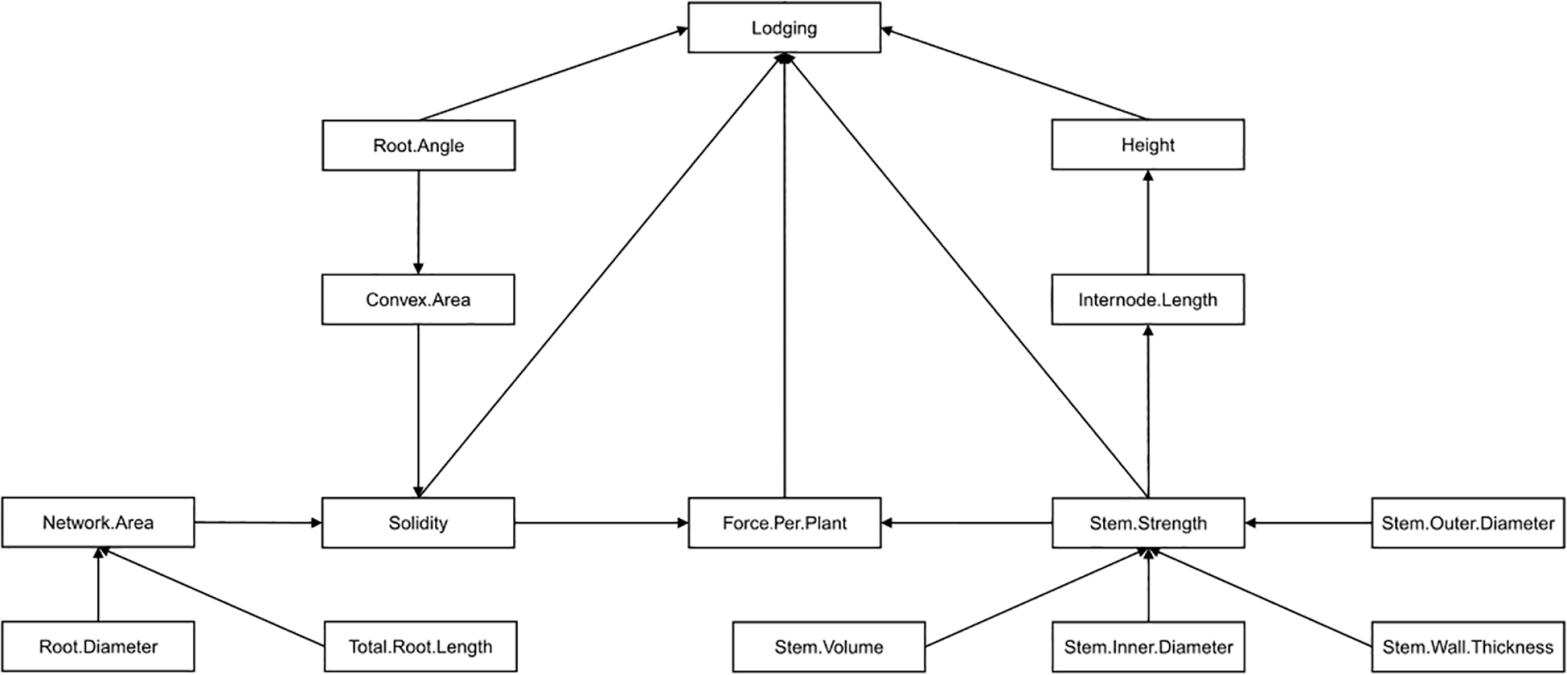
The initial hypothesized structural equation model describing the influence of 14 plant, stem and root traits on barley lodging. Traits were included in the model based on significant (P< 0.05) correlations to lodging observed in this study, significant correlations to one another observed in this study, or identified as relevant to lodging in other studies.

To build a structural equation model representing trait relationships to lodging, an initial model which hypothesized the causal relationships (i.e. paths) amongst variables and in relation to lodging was built (**Figure 3**). The inclusion of, and relationships between, variables included in the initial model was based on both first-hand knowledge and relevant literature. Internode length was considered a component of height so a path from internode length to height was included. Stem strength was included as a path to internode length and as a direct path lodging because of its strong correlation to internode length in this study and its importance to lodging resistance based on past studies (Berry et al., 2006). Stem size traits were included as direct paths to stem strength due to their strong correlations found in this study and based on their connection to stem strength in past research (Ookawa et al., 2010; Yao et al., 2011). Root network area and convex area were included as direct paths to solidity since these are the two component traits used to calculate solidity, the first describing the physical size of the roots and the second describing the outer shape of the root system within the soil. Root length and root diameter were included as direct paths to network area since these two traits were positively correlated to network area and collectively would relate to the physical size of the root system. A path from root angle to convex area was included due to their positive correlation and the idea that root angle is key to setting the overall outer shape of the root system. Finally, despite no direction correlation to lodging, force per plant was included as a direct path to lodging based on past literature indicating the connection between these two traits (Berry et al., 2003b). A direct path to force per plant from both solidity and internode strength was included since both root lodging (as influenced by solidity) and stem lodging (as influenced by stem strength) can be captured in the force per plant measurement (Berry et al., 2003b). An iterative process followed where the initial model was assessed by calculating and comparing the covariance matrix (known as the model-implied covariance matrix) associated with the variables in the initial model to the actual covariance matrix calculated for the variables using the measured data. The model-implied covariance matrix associated with the initial model, or subsequent models created by removing or adding paths in the initial model, were compared and when it was not significantly different (i.e. P > 0.05) from the actual covariance matrix, then the model was considered to accurately represent the data.

**Figure 4:**
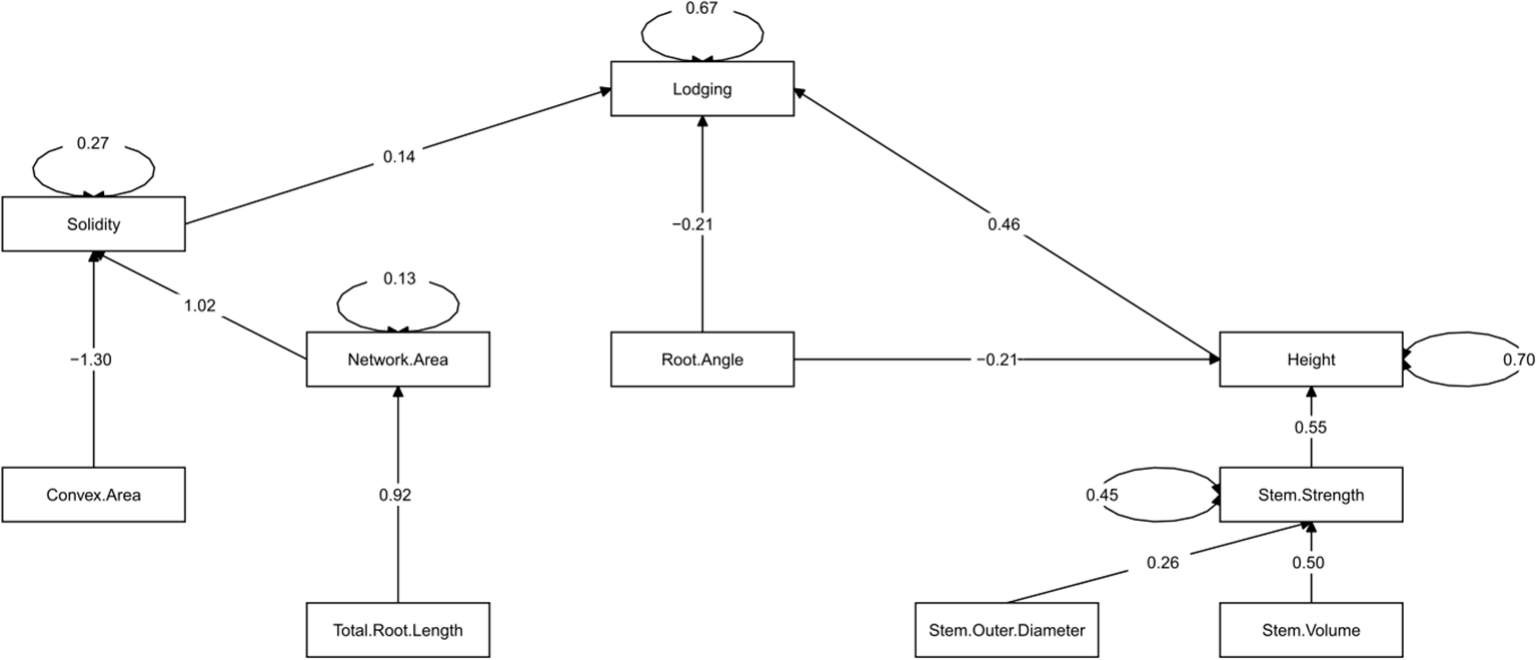
The final fitted structural equation model describing the influence of nine plant, stem and root traits on barley lodging. Causal relationships are indicated by path arrows with the predictor trait indicated at the tail of the arrow and the outcome trait at the head of the arrow. The number associated with each path arrow indicates the standardized effect estimate of the predictor trait on the outcome trait. Only significant paths (P< 0.05) are displayed. A circle created by two arrows indicates the unaccounted variation associated with the outcome trait.

The iterative process of removing and including different sets of traits (paths) ultimately assessed 10 different models, where a best-fitting final model was identified with P = 0.10, CFI = 0.990, and SRMR = 0.04 (**Figure 4**; **Table 4**). Direct paths to lodging, consistent with observed correlations (**Figure 2**) were identified for height, root angle and solidity (r = 0.46, -0.21, 0.14, respectively). As with the correlation analysis, force per plant did not produce a significant path to lodging and therefore it was removed from the model, along with paths from solidity and internode strength to force per plant. The inclusion of an internode length to height path did not permit the creation of a good fitting model, but once removed the subsequent path from stem strength to height improved model fit. However, the direct path from stem strength to lodging was not significant and was removed from the final model. Among the four stem size traits included in the initial model, it was determined that stem outer diameter and stem volume were sufficient to capture the influence of these traits on stem strength, ultimately contributing to the final model. The inclusion of stem outer diameter and stem volume in the model is consistent with their correlation to stem strength observed in this study. A path from convex area to solidity remained in the final model which was consistent with the correlation observed between these two traits. Interestingly, the network area to solidity path was also significant in the final model which differed from the correlation analysis in which no significant correlation was observed between these traits. Between the total root length and root diameter paths to network area that were included in the initial model, only total root length remained as a significant path which was consistent with the correlation observed between these traits. The path from root angle to convex area proved to be not significant, but it was determined that a path from root angle to height was an important component to creating a good fitting model. This differed from the correlation analysis in which no significant correlation was observed between root angle and height.

**Table 4:** Summary of the direct and indirect paths associated with the nine traits used in the final fitted structural equation model to describe barley lodging.

Path	Effect estimate	Std. error	P-value
Direct paths			
Solidity → Lodging	0.136	0.057	0.018
Height → Lodging	0.462	0.058	0.000
Root Angle → Lodging	-0.214	0.061	0.000
Convex Area → Solidity	-1.304	0.052	0.000
Network Area → Solidity	1.022	0.052	0.000
Total Root Length → Network Area	0.916	0.025	0.000
Stem Outer Diameter → Internode Strength	0.258	0.073	0.000
Stem Volume → Internode Strength	0.504	0.073	0.000
Root Angle → Height	-0.206	0.062	0.001
Internode Strength → Height	0.546	0.061	0.000
Indirect paths			
Convex Area → Solidity → Lodging	-0.177	0.075	0.018
Network Area→ Solidity → Lodging	0.139	0.059	0.018
Internode Strength → Height → Lodging	0.252	0.042	0.000
Root Angle → Height → Lodging	-0.113	0.039	0.004
Overall indirect relationships	0.101	0.049	0.040

The effect estimate is the strength of the causal relationship between the initial and final variable in a path and are standardized to allow direct comparison between paths. Only significant paths (P< 0.05) were retained in the final model.

## 4 Discussion

Crop lodging resulting from either stem breakage or displaced root systems leads to challenging harvest conditions, reduced yield, lower grain quality and increased disease, thus diminishing profit margins for growers (Bayer Crop Science, 2021; Berry et al., 2006; Clarkson, 1981; Nakajima et al., 2008; Niu et al., 2016; Sterling et al., 2003). As such, lodging resistance is a key trait for improvement by plant breeders. Visual evaluation of lodging, either by eye or with the aid of UAVs, is the most common method to assess lodging, but is reliant on lodging to be present in a particular field environment which is frequently not the case. Methodologies to assess lodging risk in the absence of natural lodging have focused on: (1) the development of mechanical tools to assess the forces (resistance) required to push over stems, and (2) the identification of shoot and root traits which are most influential to lodging resistance (Berry et al., 2006; Erndwein et al., 2020). In this study, evaluation of a diverse set of 13 barley genotypes for 18 above and below-ground traits revealed traits that were significantly correlated to lodging and important in a SEM for lodging. The most relevant included the above-ground trait, plant height, and the below-ground traits, crown root angle and root system solidity. To move forward, a consideration of how these traits relate to lodging, in the context of the literature, is important for the ultimate success of defining traits and methods for generating cultivars with improved resistance.

### 4.1 Height is the primary above-ground driver of barley lodging resistance

Plant height, a well-documented factor related to lodging, exhibited a positive correlation with lodging (r = 0.37) with shorter plants being more lodging resistant. This result was consistent with Berry et al. (2006) who showed a 12% increase in lodging for every 25% increase in height. In cereals such as wheat and rice, introduction of the semi-dwarf trait during the ‘Green-Revolution’ not only promoted significant increases in harvest index but also increased lodging resistance by decreasing shoot (stem) leverage forces (Berry et al., 2004; Hedden, 2003). In barley, the introduction of semi-dwarf mutations to decrease lodging through reduced height is also generally associated with more robust culm (stem) phenotypes (Braumann et al., 2018; Kristensen et al., 2016). However, in wheat, it has been demonstrated that reducing height below an optimum of approximately 70-90 cm can reduce grain yield due to sub-optimal source-sink balance and light distribution to the canopy (Reynolds et al., 2009; Slafer et al., 2023). In barley, dwarf genotypes (as compared to semi-dwarf) often exhibit later maturity, increased disease susceptibility, and reduced malt quality (Mickelson and Rasmusson, 1994). Madić et al. (2016) reported that an optimal height for spring malting barley in Serbia has been reached (at about 80 cm) with winter malt barley heights of between 90-100 cm having the potential for further reductions to promote lodging resistance. In this study, genotypes with a lodging score less than four were on average 71 cm, whereas when the lodging score was greater than 4, the average height was 79 cm. The high heritability associated with plant height, along with its ease of measurement, make it a useful and suitable trait for early generation selection in breeding programs. It should also be noted that for forage barley, height can be an important trait driving dry matter (Gill et al., 2013), thus other traits would be important for reducing lodging risk if height compromises forage yields.

Stem strength has been regarded as an important trait to improve lodging resistance in wheat, barley, and canola (Berry et al., 2006; Hai et al., 2005; Wu and Ma, 2018). However, in the current study there was no direct correlation between stem strength and lodging resistance, suggesting that the stem strength variation present in western Canadian barley is sufficient to resist lodging and stem-based failure within the study’s environmental conditions and agronomic inputs. This is consistent with the observation that selections for shorter, more rigid stems which have occurred in breeding programs (over decades) have resulted in less stem-based lodging (Crook and Ennos, 1993) and placed more emphasis on the root system as the source of lodging (Crook and Ennos, 1994). The importance of stem strength, at least in the context of the genotypes used in this study, is to support taller plants, as seen by the strong positive path in the SEM. The positive correlation between these traits differs from reported relationships of shorter plants having stronger stems (Niu et al., 2012), a relationship which is also the basis of PGR effectiveness (Kamran et al., 2018a, 2018; Niu et al., 2022). The inclusion of tall forage barley genotypes in this study (i.e. CDC Maverick and AB Cattlelac) in which tallness is intentionally bred into these varieties to improve biomass is likely the reason for the positive correlation observed in this study as these two genotypes were the tallest and also had the highest stem strengths. Additionally, the observation that AB Cattlelac showed good lodging tolerance while CDC Maverick was the poorest further highlights that stem strength is not a predictive trait for lodging tolerance in this set of germplasm. The SEM also showed that both stem outer diameter and stem volume had positive paths towards stem strength. The importance of both stem diameter and cell wall thickness (which is a component of the stem volume calculation in this study) has been noted previously in wheat and rice (Ookawa et al., 2010; Piñera-Chavez et al., 2016a; Yao et al., 2011), thus indicating that these relationships also hold true in barley.

Root lodging is usually reported as the predominant form of lodging, especially in conditions of high soil moisture, but stem lodging can still be prevalent especially in conditions of high soil nitrogen (Crook and Ennos, 1994; Berry et al., 2003a; Wu et al., 2019). The priority for trait selections for stem versus root lodging resistance can vary for breeding programs depending on the production environment, agronomic inputs (e.g. PGRs), and breeding germplasm available. For example, in the Yellow-Huai River region of China, an initial wheat breeding target of reducing plant height transitioned to focus on improving stem strength (Zhang et al., 2020). The importance of trait selections to improve barley stem strength have recently been documented (Gianinetti and Baronchelli, 2024; Jaff and Jarvis, 2021). In comparison to wheat, it has been shown that barley cultivars typically have lower stem failure moment and flexural rigidity (Berry et al., 2006; Gianinetti and Baronchelli, 2024). Thus, there is perhaps further potential to improve stem strength by improving traits like stem diameter as has been accomplished in other cereals (Niu et al., 2022), although priority traits for western Canadian environments would target root anchorage improvement as discussed below.

The force required to push over a collection of stems has been investigated in relation to lodging resistance. For example, Berry et al. (2003b) demonstrated that reduced stem and root lodging in winter wheat was correlated to a greater amount of force required to push over a collection of stems. In addition, Mangin et al. (2022) showed that agronomic practices like lower plant density, split nitrogen treatments, and plant growth regulator application could also increase stem strength (pushing force), thus supplementing traits like stem wall thickness (Kashiwagi et al., 2008) or root anchorage traits like root spread, angle, length and volume (Berry et al., 2006; Wu and Ma, 2018; Zhang et al., 2022) that are correlated with pushing resistance. However, Heuschele et al. (2020) reported the opposite relationship between pushing force and lodging, with lower pushing force values associated with lodging resistant barley, oat and wheat varieties. In the current study, force per plant was correlated with familiar traits such as height, stem strength, root length and solidity (which captures features of the root system similar to volume), but neither the correlation analysis nor the SEM found that force per plant was relevant to lodging. One explanation for the lack of relationship between force per plant and lodging would be that once stem strength reaches the threshold to protect plants from stem failure, then flexibility becomes a more important trait that would allow barley plants to flex and recover (as opposed to acting as a rigid object against wind-imposed forces). As noted by Wu and Ma (2018), plants with strong, less flexible stems that can prevent stem buckling may become susceptible to root anchorage failure as the leverage exerted on a plant by the wind is translated to the root system. Greater flexibility of barley stems, in comparison to wheat stems, has been noted by Berry et al. (2006). Stems of barley landraces with thinner diameters have been shown to flex and curve without any stem failure with the spike eventually touching the ground (Jaff, 2019). As the current study was not able to measure shoot flexibility or elasticity, this would be a future point of focus.

### 4.2 Crown root angle and solidity are key below-ground traits impacting barley lodging resistance

Crown root angle emerged as an important trait associated with barley lodging in the current study. This is the first report to demonstrate a direct relationship between root angle and lodging in barley. In corn, increased brace root angle was also observed to enhance lodging resistance (Brune et al., 2017; Shao et al., 2021; Sparks, 2022; Zhang et al., 2022). Root angle has also been shown to be relevant to wheat root anchorage strength (Crook and Ennos, 1994), with root anchorage strength reported to be an important component of lodging resistance in wheat, barley and canola (Berry et al., 2006; Piñera-Chavez et al., 2016b; Wu and Ma, 2018). Crown root angle was a strong driver of root plate spread in the current study (r = 0.72) and previous studies have observed a correlation between greater root plate spread and lodging resistance in wheat and corn (Berry et al., 2003c; Hostetler et al., 2022). Despite the strong correlation between crown root angle and root plate spread, no correlation between root plate spread and lodging was observed. The importance of root angle to lodging was also confirmed in the SEM, with a direct negative path between the two traits being necessary for the final model. It was also interesting to note that root angle displayed an indirect path to lodging, via a negative path to height. This path was required to produce a good fitting model and indicated that narrower root systems were associated with taller plants. This relationship may hint at an underlying genetic basis, for example, Bai et al. (2013) identified overlapping QTL in wheat that controlled plant height, as well as root volume and root length. Identifying two paths to lodging from root angle emphasizes the importance of this trait to lodging resistance. Notably, root angles were highly heritable not only in this study, but also in sorghum and corn (Lopez et al., 2017; Wang et al., 2021). Thus, root angle would be a suitable selection target for lodging resistance at early stages of breeding programs, potentially by using controlled environments facilities or greenhouses. For example, wheat seedling root traits, which can act as surrogate predictors of important adult root traits, have been assessed using growth pouches (Richard et al., 2015).

Root system solidity, which describes the ratio between network area (i.e. total root tissue) and convex area (i.e. smallest polygon containing the root system), exhibited correlations to several root systems traits such as root angle, root spread, root system width and as expected, convex area. Such correlations between various root traits and solidity have been observed previously in wheat, soybean, strawberries, Arabidopsis, and alfalfa (Cockerton et al., 2020; Deja-Muylle et al., 2022; Falk et al., 2020; Mattupalli et al., 2019; Shao et al., 2021). However, the most relevant and novel correlation that solidity displayed was to lodging (r = 0.62), a finding that no prior studies have described.

Solidity was also an important component of the SEM. A direct positive path from solidity to lodging was observed, while strong paths from network area and convex area to solidity were important contributors to the model. The finding that solidity represents a central trait describing root system architecture and the distribution of biomass across soil space in both the correlation analysis and SEM was not surprising. Solidity has been described as a measure of soil exploration efficiency comparing compact versus exploratory architectures and reflects the trade-off between foraging in-place behavior and space exploration (Bontpart et al., 2020; Mattupalli et al., 2019). In the present study, cultivars with a wider root angle showed correlations to both convex area and solidity (r = 0.43 and -0.57, respectively), indicting that root angle plays an important role in setting the overall outer dimensions of the root system (or soil volume the root system occupies). Wider root angle promoted lower solidity and a more exploratory architecture with increased root plate spread, hence why lower solidity is related to lodging resistance. The strong paths from convex area and network area to solidity captured by the SEM reflect the traits used to calculate solidity, while the strong path from total root length to network area connect two traits related to the size of a root system. Although solidity has not been identified previously as being related to lodging, total root length (Wu and Ma, 2018) and root volume (Wu and Ma, 2018; Zhang et al., 2022) have been associated with increased lodging resistance in maize and canola.

The correlation between solidity and lodging, the inclusion of solidity in the SEM, and the moderate heritability of solidity positions this trait as a promising candidate for barley breeders to use for selections. Moderate to high heritability values for solidity have been reported in corn and *Brachypodium* (Ingram et al., 2012; Shao et al., 2021). Findings in corn and rice have demonstrated that solidity can remain relatively constant over time as a means of controlling root density and maintaining the ratio of root mass to root system size and the space it occupies (Topp et al., 2013; Zurek et al., 2015). However, it should be noted that differences in soil fertility (e.g., phosphorus or nitrogen) have been shown to impact solidity in sorghum and canola and thus can influence heritability (Parra-Londono et al., 2018; Qin et al., 2019). Thus, future assessments of solidity across different soil fertility levels are warranted.

### 4.3 Summary

Among the 18 traits evaluated in this study, plant height, root angle and solidity were consistently observed in both correlation analysis and SEMs to influence lodging. Importantly, this study is the first to establish in barley that root angle is an important trait relevant to lodging resistance, and is the first report that root system solidity is also a key lodging resistance trait. The high heritability of both plant height and root angle indicates they could be incorporated into earlier generation selection strategies by barley breeders while solidity, with its moderate heritability and somewhat lower throughput, could be utilized at later generations when lower numbers of genotypes are being assessed. Predictive modelling for root traits using simple regression or machine learning algorithms could be used to rigorously assess potential parental germplasm prior to crosses and expansion into large-scale field trials within a breeding program. Exploring the possibility of rapidly assessing root traits, such as angle or solidity, in a high-throughput indoor environment is a future goal towards increasing both time and cost efficiencies for lodging selections. It was also revealed that stem-related traits, such as stem strength, diameter or wall thickness, and plant bending resistance (i.e. force per plant), which have been shown to influence lodging in other barley growing regions or in other crops, demonstrated no significant predictive value for barley lodging resistance in western Canadian environments.

This study confirmed previous assertions that decreased height and increased root spread, demonstrated here through increased root angle, contribute to lodging resistance and lends credence to the use of root crown imaging to assess the relationship of root architectural features with complex phenomenon such as lodging. Collectively, this study offers breeders avenues to increase lodging resistance especially in the absence of visible lodging in field trials during germplasm development.

## Data Availability

The raw data supporting the conclusions of this article will be made available by the authors, without undue reservation.
